# Efforts to Prevent the Neglect of Traditional Indigenous Medicine: Policy Insights From Bolivia

**DOI:** 10.1002/hpm.70126

**Published:** 2026-08-12

**Authors:** Carlos Rosas‐Jiménez, Roberto Tola, Melina Flores, Sonia Cruz, Gonzalo Huanca, Cynthia Lokker, Bonny Ibhawoh, Lisa Schwartz

**Affiliations:** ^1^ Mary Heersink School of Global Health and Social Medicine McMaster University Hamilton Ontario Canada; ^2^ Aymara Indigenous Community Bolivia Bolivia; ^3^ Department of Health Research Methods Evidence & Impact McMaster University Hamilton Ontario Canada; ^4^ Centre for Human Rights and Restorative Justice McMaster University Hamilton Ontario Canada

**Keywords:** Bolivia, health policy, holistic health, indigenous, intercultural medicine, traditional medicine

## Abstract

Traditional Indigenous medicine has existed for centuries, but only recently has it obtained international recognition. Some countries have been working on the integration of traditional Indigenous healing practices in their national health systems. For example, Bolivia has taken significant steps to prevent the neglect of traditional Indigenous medicine over the last 40+ years, a process that continues today through several policies supported by the national government. The aim of this perspective article was to discuss efforts to prevent the neglect of traditional Indigenous medicine in Bolivia. Through analysis of 12 legal documents, including the Constitution of 2009, decrees, laws and resolutions, we categorised the efforts in 5 points: acknowledging the past, making radical changes to the political constitution, adopting a new approach to health and wellness, regulating traditional medicine, and protecting the environment.

## Highlights

- Respecting Indigenous knowledge involves respecting Indigenous people.
- Valuing the past is necessary to preserve traditional Indigenous medicine.
- Changes in the political Constitution can bring further necessary changes.
- There is no traditional Indigenous medicine without environmental protection.

## 1 Introduction

Throughout history, Indigenous peoples worldwide have accumulated a wealth of knowledge spanning various facets of life. They have thrived for centuries, and some communities still embrace ways of life that have persisted for generations. The continued existence of these communities and the preservation of their environments serve as a testament to the resilience and strength of their way of life. Despite their wisdom and resilience, Indigenous peoples have been oppressed and discriminated against in many countries around the world. Colonisation has led to the social oppression of Indigenous peoples and the exclusion of their traditional knowledge.

The lack of humility of those involved in the colonisation process has led to the marginalisation of Indigenous peoples for several centuries. This lack of humility prevents us from being open‐minded and exploring how Indigenous healing practices work and understanding the fundamental elements of the framework on which they are based.

Efforts to understand and promote respect for Indigenous traditions and knowledge increased, especially at the end of the twentieth century. In 1978, the Alma Ata Declaration acknowledged traditional medicine and its practitioners as resources for achieving health for all by the year 2000 [1]. Later on, in 2007 the United Nations General Assembly adopted the Declaration on the Rights of Indigenous Peoples (UNDRIP) [2] making significant contributions to the protection of Indigenous traditions and knowledge. More recently, on one of its latest reports, the World Health Organization (WHO) describes a national framework for traditional and complementary medicine (T&CM), product regulation, practices and practitioners, the challenges faced by countries, and the situation regarding T&CM in some countries around the world [3].

The main goal of this article was to discuss efforts to prevent the neglect of traditional Indigenous medicine in Bolivia. Key points include acknowledging the past, making radical changes to the political constitution, adopting a new approach to health and wellness, regulating traditional medicine, and protecting the environment (see Figure 1). A documentary analysis of some relevant policies regulating traditional medicine and Indigenous health issues in Bolivia was conducted to achieve this goal (see Table 1). Understanding the Bolivian case is essential for considering it as a model for recognising and appreciating the holistic healing frameworks of Indigenous peoples, as well as respecting their human rights, knowledge, and traditions.

**FIGURE 1:**
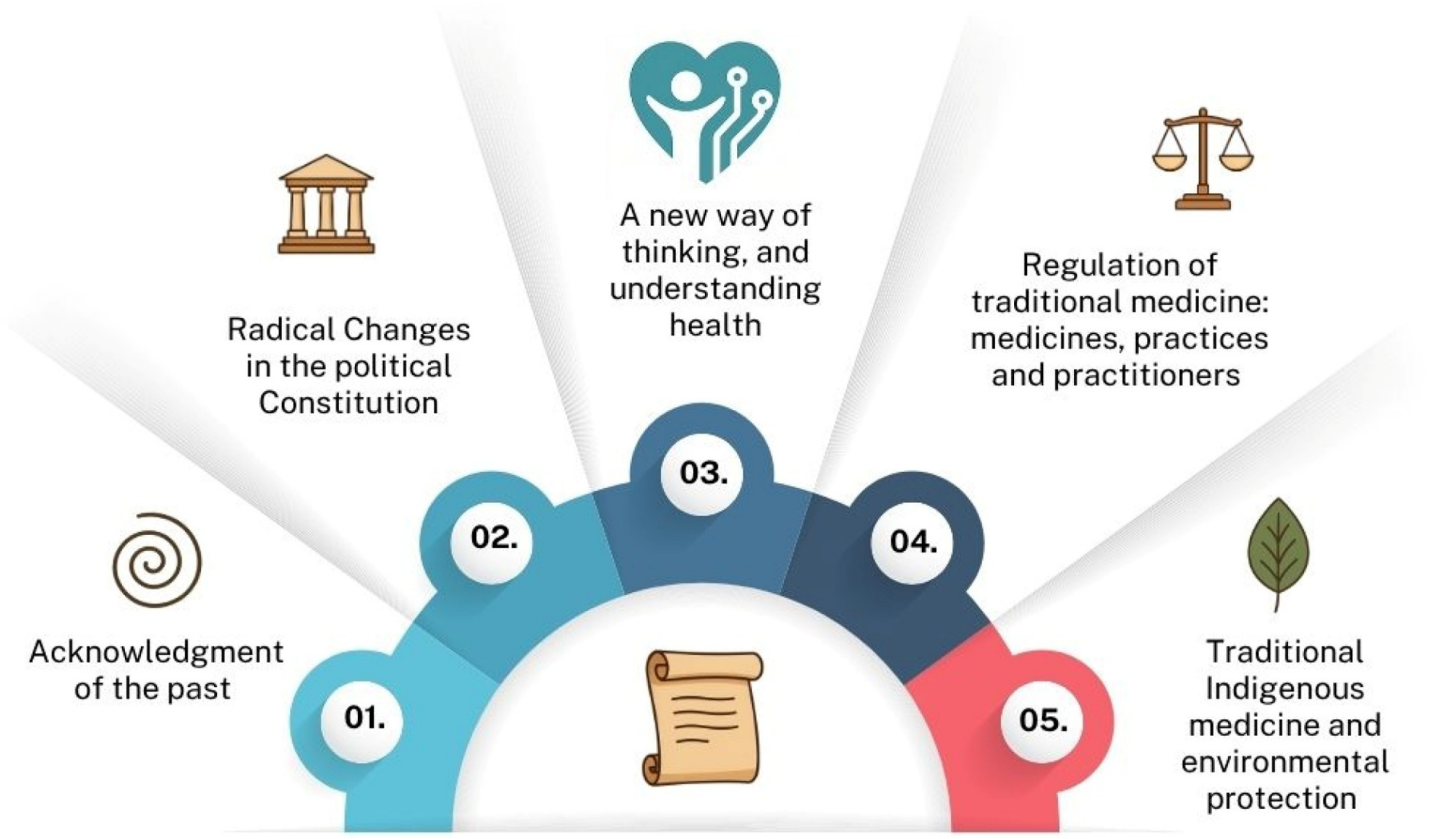
Efforts to prevent the neglect of traditional Indigenous medicine: Insights from Bolivia.

**TABLE 1:** List of the included documents organised in chronological order.

#	Year	Policy	Links
1	1984	Supreme resolution no. 198771	[4]
2	1987	Resolution 0231	[5]
3	1987	Law 0928	[5]
4	1992	Law no. 1333	[6]
5	1996	Supreme decree no. 24676	[7]
6	1996	Law 1737	[8]
7	1998	Supreme decree 25235	[9]
8	2008	Supreme decree 29601	[10]
9	2009	Bolivian Constitution	[11]
10	2013	Law 459	[12]
11	2015	Supreme decree 2436	[12]
12	2020	Supreme decree no. 4257	[13]

### 1.1 Acknowledgement of the Past: Indigenous Knowledge and Traditional Medicine

In a report sponsored by the Spanish Embassy in Bolivia and based on historical documents, Baixeras [14] determined what integration exists in Bolivia between traditional empirical medical practices and modern scientific medicine. The report presents a summary of the main publications on traditional medicine in Bolivia in chronological order from 1553 to 2004 [14]; the information has been classified and presented in Figure 2. These documents do not include government policies. Most of the documents were scientific reports and descriptions of the vegetation and natural resources found in Bolivia and some of them included explanations of their medicinal value (See Table 2). However, the legal recognition of traditional medicine began in the decade of the 1980s.

**FIGURE 2:**
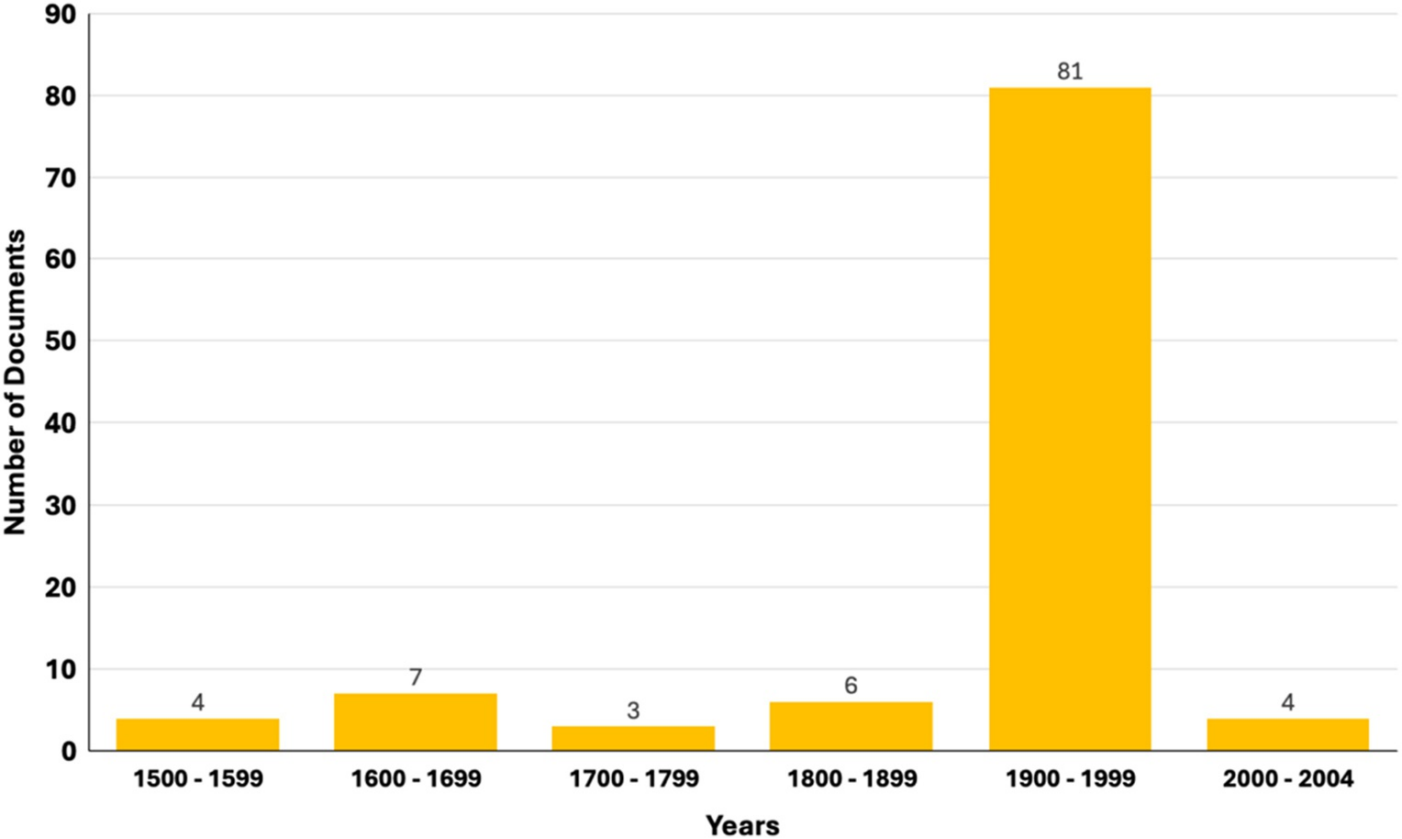
Number of relevant documents related to Bolivian traditional medicine based on Baixeras [14].

**TABLE 2:** Examples of relevant historical documents related to traditional medicine in Bolivia that were published within the last five centuries.

Year	Author	Title
1565	Nicolás Monarde	*Historia medicinal de las cosas que se traen de las Indias Occidentales que sirven en medicina* (Medicinal History of the Things Brought from the West Indies that are Useful in Medicine)
1627	Gregorio de Loza Vila y Palomares	*Cosas Medicinales* (Medicinal Things)
1760	Delgar	*Terapéutica indígena boliviana* (Bolivian Indigenous Therapeutics)
1889	Nicanor Iturralde and Eugenio Girault	*Clasificación de las Plantas Medicinales en la Farmacología Callahuaya o sea de los Indios Curanderos Aimaras* (Classification of Medicinal Plants in the Callahuaya Pharmacology or that of the Aymara Indian Healers)
1950	P. Villarroel	*Manual de Plantas Alimenticias, Medicinales e Industriales de Bolivia* (Handbook of Food, Medicinal and Industrial Plants of Bolivia)

Three events that favoured traditional medicine occurred in the 1980s: the promulgation of the Supreme Resolution No. 198771 of 1984; the issuance of Resolution 0231 of 1987; and the promulgation of Law 0928 of 1987. In 1984, the Supreme Resolution No. 198771 officially recognised SOBOMETRA as the first organization of traditional doctors established at the national level [15]. SOBOMETRA is an NGO dedicated to the investigation of ancestral knowledge on the use of medicinal plants and other healing practices, for and in Indigenous and peasant communities, so that people begin to use and practice this type of medicine as an alternative to the state's neglect in the area of public health [16]. Three years later, the Ministry of Social Welfare and Public Health, considering that it was necessary to regulate everything related to the practice of naturopathic‐traditional medicine in Bolivia and to protect and safeguard the health of all, issued regulations for traditional medicine practice through Resolution 0231 of 1987 (See Table 3) [5].

**TABLE 3:** Details of resolution no. 0231 of 1987.

Characteristics	Detail
Issuing body (name in Spanish)	Ministerio de provisión social y salud pública (Ministry of social provision and public health)
Issued	1987
People in charge of execution and compliance	General director of public health and the directors of the health units
Referral to traditional medicine (terms used)	Medicina naturista‐tradicional (Naturopathic‐traditional medicine)
Definition of traditional medicine	Traditional medicine is a set of practices, resources and diverse procedures that have been used ancestrally by the native inhabitants of our country.
Definition of other related terms	Traditional naturopathic medicine from Bolivia: It is the native practice that uses some elements of the three kingdoms of nature; without having been previously subjected to manipulations or procedures that previously modify their essence (animal, plant or mineral) learnt by practitioners who have continued their learning from generation to generation by oral tradition
Objective of traditional medicine	For the treatment of ailments.
Use of traditional medicine throughout the country	It is widespread, particularly in rural areas.
Main components of traditional medicine	1. The healer, who makes a truly magic‐therapeutic ceremony, which has great influence on the sick person. 2. Resources for the treatment, especially plants and herbs. Many of them have curative effects, but there are others of debatable usefulness, or even potentially harmful.
Topics discussed	1. Requirements for the practice of traditional medicine. 2. Duties and obligations of practitioners of traditional naturopathic medicine in Bolivia. 3. Records and control of facilities, patients, use of substances, scientific studies, commercialisation, 4. Training of practitioners. 5. Requirements for the opening of establishments for the practice of traditional naturopathic medicine in Bolivia. 6. Tariffs regulated by the health authority. 7. Associations of traditional naturopathic medicine in Bolivia.
Other responsible stakeholders	The secretariat of social security and public health and the bolivian society of traditional medicine (SOBOMETRA)
Reference to international global health documents	Alma Ata declaration: ‘Relies, at local and referral levels, on health workers, including physicians, nurses, midwives, auxiliaries and community workers as applicable, as well as traditional practitioners as needed, suitably trained socially and technically to work as a health team and to respond to the expressed health needs of the community’.

### 1.2 Radical Changes in the Political Constitution

The Bolivian constitutional system is a product of changes, the result of adjustments to historical realities, occurring within the framework of an always weak democracy, interrupted by repeated coups d'état and with unquestionable deficiencies in form and substance of the Political Constitution of the Bolivian State [17]. Bolivia's constitutional history dates back to the first Political Constitution or ‘Bolivarian Constitution’, draughted at the dawn of independence on August 6, 1825, and to the founding of the Republic by the Liberator Simón Bolívar. Although the reform process has always been complex, the country has had 19 constitutional texts, that is, 1831, 1834, 1839, 1843, 1851, 1861, 1868, 1871, 1878, 1880, 1899, 1931, 1938, 1945, 1947, 1961, 1967, 1994, and 2004 [17].

Although repeated changes to the constitution have not altered its original essence [17], in 1994, the first article of the Bolivian Constitution was modified, introducing, in the points referring to the characterisation of the country, the multiethnic and pluricultural aspects, which include Indigenous communities, in contrast to the wording adopted by the various Bolivian constitutional texts from 1878 to 1967 in which Bolivia proclaimed itself as a unitary, democratic and representative republic [18]. In this way, Indigenous peoples gained more recognition and the groundwork was laid for the radical changes to the Constitution in 2009.

Developing the new Constitution was a long process that required the involvement of many actors, where Indigenous peoples had a very important role. As Doyle [19] describes it, ‘the resulting 2009 Constitution is one of the most radical legal documents in history, expressing full recognition of non‐state systems of law, the legal personhood of the Mother Earth and Indigenous values as guiding national principles. Most importantly it codifies decolonisation into law and establishes the frameworks through which the state and wider society are to be transformed to undo the systematic oppression suffered by Bolivia's Indigenous majority population’ [19].

The Constitution is characterised by the inclusion of the axiological content of Indigenous cultures, including their values and principles; for example, more than 30 articles directly relate to the rural Indigenous population and correspond to their economic, civic, political, social, cultural, territorial and environmental rights, as well as their Indigenous jurisdiction and autonomy etc. [20]. Furthermore, for example, numeral three of article 18 of the Bolivian Constitution of 2009, which states that the health system will be intercultural: ‘El sistema único de salud será universal, gratuito, equitativo, intracultural, intercultural, participativo, con calidad, calidez y control social’ (The single health system will be universal, free, equitable, intracultural, intercultural, participatory, with quality, warmth, and social control) [10].

In addition, the 2009 Constitution introduced the concept of ‘Living Well’ (*‘Vivir bien’* in Spanish) [10]. While Ecuador uses the term ‘Good Living’ (‘Buen Vivir’ in Spanish) known in *Kichwa* (Quechua) as *Sumak Kawsay*; Bolivia uses the term ‘Living Well’, whose official Aymara translation is *Suma Qamaña* [20]. ‘Living well’ appears seven times in the Constitution of Bolivia [21]. *Suma Qamaña*, that is, Living Well, combines the idea of mutual support and solidarity with peaceful social coexistence and material well‐being [21]. Consequently, Living Well is, in essence, a meta‐value to which other more common values, such as, social equity, equality and inclusion must be subordinate [21]. This subordination may only be possible if ‘Living Well’ is not an abstract category, but rather if it comes from the Indigenous expression *Suma Qamaña*. Subsequent national policies have been added to Bolivian legislation, making real a hopeful process of change towards Living Well, and bringing about changes and reforms that benefit the populations [20].

### 1.3 A Way of Thinking, and Understanding Health That Considers Indigenous Knowledge: The SAFCI Model

One year before the enactment of the 2009 political constitution, a significant step in the recognition, integration, and implementation of traditional medicine was taken. The Supreme Decree 29601 of 2008 established the SAFCI model (‘Salud Familiar Comunitaria Intercultural’, in English ‘Intercultural Community Family Health’) [11]. Conceived as a new way of thinking, feeling, understanding, and practicing health, the SAFCI model brings together the health personnel and traditional doctors of Indigenous and native peasant communities with individuals, families, communities, Mother Earth and the cosmos, based on their organizations' participation in management, social control, and comprehensive intercultural healthcare [22].

The objectives of the SAFCI model (See Figure 3) are to contribute to the elimination of social health exclusion (translated as effective access to comprehensive health services); to vindicate, strengthen and deepen effective community participation in decision making in participatory management and social control; and provide health services that take into account the person, family and community [22]. This model is based on four principles: community participation, integrality, interculturality and intersectoriality, which make it a practical theoretical model [22]. In that way, the SAFCI model was designed not only to reduce social exclusion in health, but also to contribute to the establishment of regional, departmental, and municipal workshops as platforms for the recovery and exchange of knowledge and practices, serving to plan policies that foster collaboration and complementarity between traditional medicine and biomedicine [24].

**FIGURE 3:**
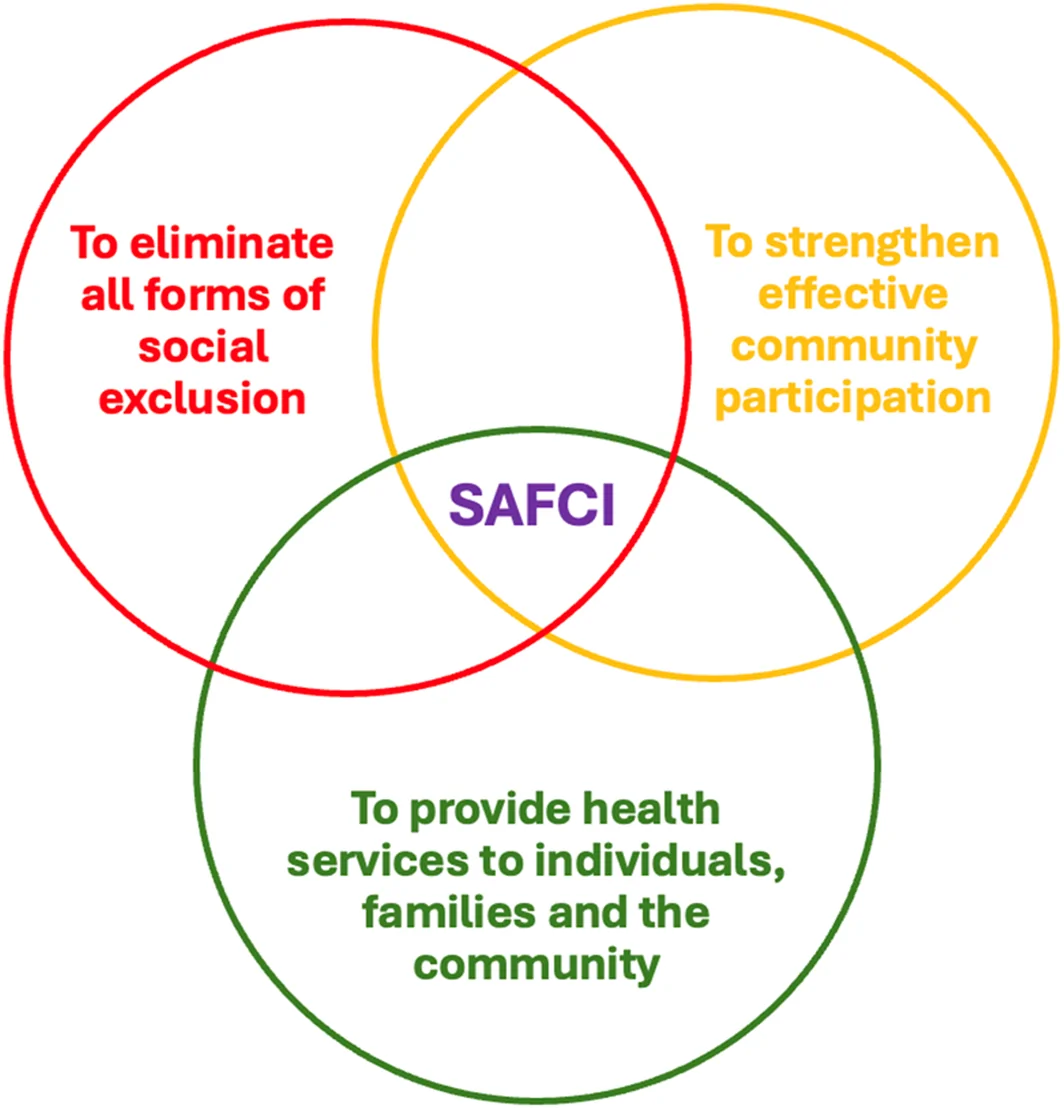
Graphic representation of the main objectives of the SAFCI model based on Polo Andrade [23].

Five years after the SAFCI model was established by the Supreme Decree 29601 of 2008, positive results were documented in three main areas around the country [24]: (1) SAFCI Mobile teams: 44 teams in 44 municipalities performing interdisciplinary health functions within the framework of the SAFCI Policy at the national level; (2) SAFCI specialists: 155 SAFCI specialists in 116 municipalities providing services to communities within the framework of the SAFCI Policy; (3) health professionals of the year of compulsory social service (ASSO in Spanish: Año de Servicio Social Obligatorio): 332 ASSO specialists providing community service in 84 municipalities across the country. Much more recently, a cross‐sectional observational descriptive study found that the SAFCI Model was partially applied in the seven Health Networks in Chuquisaca, Bolivia but not in tertiary care hospitals in the province [23].

The application of the SAFCI Model at the national level has encountered problems of lack of continuity in its implementation processes due to sudden changes in institutional Human Resources [25]. Furthermore, an evaluative study by the Ministry of Health in first‐level care health establishments in four Health Networks of La Paz during the 2012 and 2015 administrations found that more than 62% of the institutional staff did not know the Health Model nor the strategy for its implementation, and it was evident that more than 81% of the services under investigation had not applied the principles of intersectoriality and interculturality in the care and articulation process [25].

The SAFCI model has introduced new insights into the Bolivian health system, defining health as a process of biopsychosocial, cultural, and spiritual balance and harmony of the individual with themselves and their environment, which involves the family, the community, and nature [22]. Despite the implementation challenges in Bolivia, the SAFCI policy has emerged as a progressive, community‐driven, and ideologically motivated approach when considering health policy reforms in an international context [26]. Bernstein [26] argues that many healthcare systems around the world are dominated by neoliberal, market‐based approaches. On the contrary, he explains that ‘Bolivia's health policy, and the study of its making is thus distinctive and instructive for thinking about how alternatives emerge, and how a country with limited resources can build momentum towards an inclusive political ideology that is grounded in community participation and a sense of community ownership over health’ [26].

### 1.4 Regulation of Traditional Medicine: Medicines, Practices and Practitioners

Knowledge about traditional Indigenous medicine has been progressive, and different policies have accompanied this process. After the significant steps taken in the decade of 1980s, Law 1737 of 1996 insisted on the regulation of manufacture, elaboration, importation, commercialisation, quality control, registration, selection, acquisition, distribution, prescription and dispensation of medicines in general, including homoeopathic and natural medicinal products [8]. Two years later, Supreme Decree 25235 of 1998 was promulgated, including a full chapter dedicated to natural and traditional medicines which stipulates that natural and traditional medicines must have a specific health registration, a quality control for their commercialisation and use, as well as the verification of the identity of the raw material, which had to be certified by the National Herbarium, among other provisions [9].

The so‐called Ley 459 de Medicina Ancestral Boliviana (Bolivian Traditional Medicine Law) was promulgated in the 2010s, 26 years after SOBOMETRA was created [12]. Two years after Law 459 was issued, Supreme Decree 2436 was published to regulate it [12]. However, Elías‐Rojas [27] states that compliance with Law No. 459 and its effectiveness cannot be established and that its proposed objectives have not been achieved. According to Camacho Hinojosa [28], there is still a great deal of resistance and disdain on the part of conventional academic medical personnel towards traditional healers, midwives, and curanderos and curanderas, who continue to play a vital role in addressing the health needs of Indigenous populations in rural areas that still lack the same level of communication and care as urban areas.

More recently, an important step taken in regulating traditional medicine was the creation of the Office of the Vice Minister of Traditional Medicine and Interculturality in 2006 as part of the Ministry of Health [29]. Fourteen years later, the name was changed to Office of the Vice Minister for the Promotion, Epidemiological Surveillance, and Traditional Medicine and Interculturality, modifying and assigning it more regulatory and control functions [13].

We have categorised the obligations related to traditional medicine regulated by the Vice Ministry of Promotion, Epidemiological Surveillance, and Traditional and Intercultural Medicine, defined in Article 92 of Supreme Decree No. 4257 of June 4, 2020 (See Table 4). Having these guidelines at the level of responsibility of a deputy ministry in the Ministry of Health of a country is a good example not only of the importance given to traditional medicine but also to the need to regulate a very popular approach to human health and healing in the country.

**TABLE 4:** Obligations related to traditional medicine of the vice ministry of promotion, epidemiological surveillance, and traditional and intercultural medicine [13].

Category	Obligation
Intercultural model	— Design and propose policies for promoting traditional medicine and its integration with academic or university medicine, in the context of interculturalism and the adaptation of health services to different ethnic groups. — Design, propose, and develop a comprehensive model of care for the promotion and advancement of family and community health, respecting interculturality in communities, towns, *suyus, marcas, ayllus, tentas, capitanías*, and others. — Incorporate and develop traditional medicine practices into the health system and implement a system for registering and codifying traditional and alternative therapies, incorporating them into the national pharmacopoeia of medicines.
Practitioners	— Accredit the human resources that carry out the practice of traditional medicine and establish protocols for standards and procedures for traditional and alternative health care. — Registration and certification of traditional doctors, midwives, and naturopaths. — Develop standards and regulations for the practice of traditional medicine in the sectors of: Traditional doctors, midwives, and naturopaths.
Research	— Promote research in traditional medicine and interculturality in health services, and coordinate service networks and social networks with the practice of traditional medicine.
Intellectual property	— Implement rules and regulations regarding the protection of intellectual property of all pharmacopoeia related to traditional medicine.

### 1.5 Traditional Indigenous Medicine and Environmental Protection

Two policies in Bolivia have explicitly made a link between the protection of the environment, traditional Indigenous medicine and the role that Indigenous peoples have in the protection and management of natural resources. For example, Law No. 1333 of 1992 has not only contributed to the protection of the environment but also given Indigenous communities a more prominent role in the care, use, and exploitation of these resources, specifically in Articles 62, 64 and 78. This law encourages the development of essential protocols for the involvement of both Indigenous peoples and other traditional communities in the responsible use of renewable natural resources and the promotion of sustainable development, considering their cultural, social, and economic characteristics in the environment where they live and work [6].

Supreme Decree No. 24676 of 1996, established to regulate Decision 391 of the Commission of the Cartagena Agreement of July 22, 1996, guarantees the recognition of the rights of Indigenous peoples and rural communities as providers of the intangible component associated with genetic resources [7]. Genetic resources are understood as biological material containing genetic information that is valuable or useful [30]. This was an important measure to preserve biodiversity, which is an expression of a rich genetic patrimony.

Indigenous peoples discovered the curative power of many natural resources over the course of centuries. Since traditional Indigenous medicine is mainly based on medicinal plants, preserving natural resources and biodiversity directly contributes to preserving the raw materials of traditional Indigenous medicine.

## 2 Conclusion

The efforts to prevent the neglect of traditional Indigenous medicine in Bolivia were categorised into five points. We emphasised the importance of understanding the country's history and the evolution of its approach to natural resources and traditional medicine. Additionally, radical changes to the political constitution are a way to establish a solid foundation for the development of future laws and policies. Furthermore, adopting a new approach to health and wellness based on Indigenous traditions and knowledge has contributed to the visibility and application of traditional medicine through the SAFCI model. One way to support traditional medicine is to regulate the use of natural resources in a sustainable way. Finally, Bolivia is a notable example of respect for Indigenous traditions and knowledge, including healing practices, an example that could be applied to great benefit in other places.

## Funding

The authors have nothing to report.

## Conflicts of Interest

The authors declare no conflicts of interest.

## Data Availability

Data sharing not applicable to this article as no datasets were generated or analysed during the current study.
